# Vesicostomy button: how is it placed, in whom, and how is quality of life affected?

**DOI:** 10.1590/S1677-5538.IBJU.2018.0686

**Published:** 2019-09-02

**Authors:** Kelly J. Nast, George Chiang, Sarah Marietti

**Affiliations:** 1 University of California, San Diego, CA, USA;; 2 Rady Children’s Hospital, San Diego, CA, USA

**Keywords:** Cystostomy, Gastrostomy, Quality of Life

## Abstract

**Purpose:**

The vesicostomy button has been shown to be a safe and effective bladder management strategy for short- or medium-term use when CIC cannot be instituted. This study reports our use with the vesicostomy button, highlighting the pros and cons of its use and complications. We then compared the quality or life in patients with vesicostomy button to those performing clean intermittent catheterization.

**Materials and Methods:**

Retrospective chart review was conducted on children who had a vesicostomy button placed between 2011 and 2015. Placement was through existing vesicostomy, open or endoscopically. We then evaluated placement procedure and complications. A validated quality of life questionnaire was given to patients with vesicostomy button and to a matched cohort of patients performing clean intermittent catheterization.

**Results:**

Thirteen children have had a vesicostomy button placed at our institution in the 4 year period, ages 7 months to 18 years. Indications for placement included neurogenic bladder (5), non-neurogenic neurogenic bladder (3), and valve bladders (5). Five out of 7 placed via existing vesicostomy had leakage around button. None of the endoscopically placed buttons had leakage. Complications were minor including UTI (3), wound infection (1), and button malfunction/leakage (3). QOL was equal and preserved in patients living with vesicostomy buttons when compared to CIC.

**Conclusion:**

The vesicostomy button is an acceptable alternative to traditional vesicostomy and CIC. The morbidity of the button is quite low. Endoscopic insertion is the optimal technique. QOL is equivalent in patients with vesicostomy button and those who perform CIC.

## INTRODUCTION

The vesicostomy button is an adaptation of the gastrostomy button placed into the bladder. It was first described as a means of bladder drainage in 1996 and became popularized by our European colleagues in 2007 (1-3).The vesicostomy button is an attractive option for those patients in which clean intermittent catheterization (CIC) cannot be instituted. CIC is not feasible in some children due to anatomic variations, neurologic reasons, or pain and discomfort from catheter insertion. It has been well established that the goals of bladder management include preservation of the upper tracts, low pressure storage, and a socially acceptable means of drainage. CIC has been the gold standard owing to low risk of infection, stone formation, and erosion (4). Alternatives to CIC have been investigated including continuous indwelling Foley catheter, suprapubic tube, Mitrofanoff vesicostomy, and the vesicostomy button; each modality has unique risks and benefits and can be chosen with appropriate physician and family discussion (1).

The vesicostomy button ideally allows the patient to enjoy the benefits of urinary continence with intermittent bladder drainage without the use of a bag or catheter insertion. Placement can be done by an open technique, endoscopic technique, percutaneous technique, or insertion into an established vesicostomy tract (2). The literature has shown the vesicostomy button is a safe and effective alternative with minor complications including leakage around the button, local wound infections, and UTIs; no major complications were reported.

Improving quality of life for pediatric patients in an important consideration for pediatric urologists. Quality of life impacts care options and surgical decisions. Milliken et al. (5) postulated an improved quality of life in children managed with the vesicostomy button, owing to ease of use and discretion, however no study has objectively assessed the impact of using the vesicostomy button on quality of life.

We aim to discuss our experience with the vesicostomy button focusing on patient selection factors, technique of placement, and complications associated with the vesicostomy button. We also evaluated quality of life of the button and compared it to CIC patients. We hypothesized that quality of life would be improved with vesicostomy button use.

## MATERIALS AND METHODS

Institutional Review Board approval was obtained. A retrospective chart review was conducted on children who had a vesicostomy button placed between 2011 and 2015. Data collected included patient name, age, presentation to urology, underlying diagnoses, indication for vesicostomy button placement, length of placement at time of review, complications at the first follow-up after placement, complications within the first 30 days after placement, UTI’s 12 months before and after placement up to the date of review, creatinine before and after placement, and upper tract imaging.

**Figure 1:**
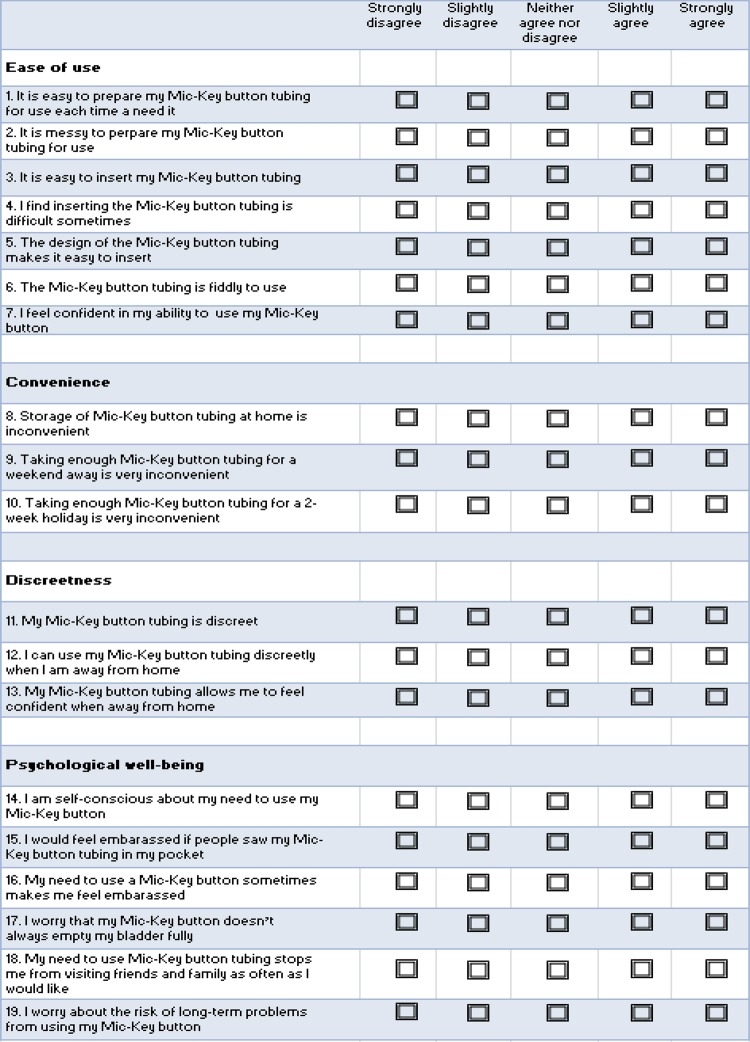
Intermittent Self-Catheterization Questionnaire (ISC-Q) adapted for Vesicostomy (Mic-Key) Button Users.

We also prospectively identified patients undergoing CIC and matched them to the vesicostomy button patients by age and gender. A validated Intermittent Self-Catheterization Questionnaire (ISC-Q) was administered to the patient or family during a scheduled clinic visit (6). The ISC-Q is a self-report survey that focuses on quality of life (Figure-1). The survey was adapted to vesicostomy button with 5 questions eliminated that relate to the single use nature of a urethral catheter. The adapted ISC-Q includes 19 items that evaluate 4 domains: ease of use, convenience, discreteness and psychological well-being. Patients and/or primary caregivers were encouraged to fill out the questionnaire depending on age and cognitive ability or the participant.

### Technique for primary placement: open technique

**Figure 2:**
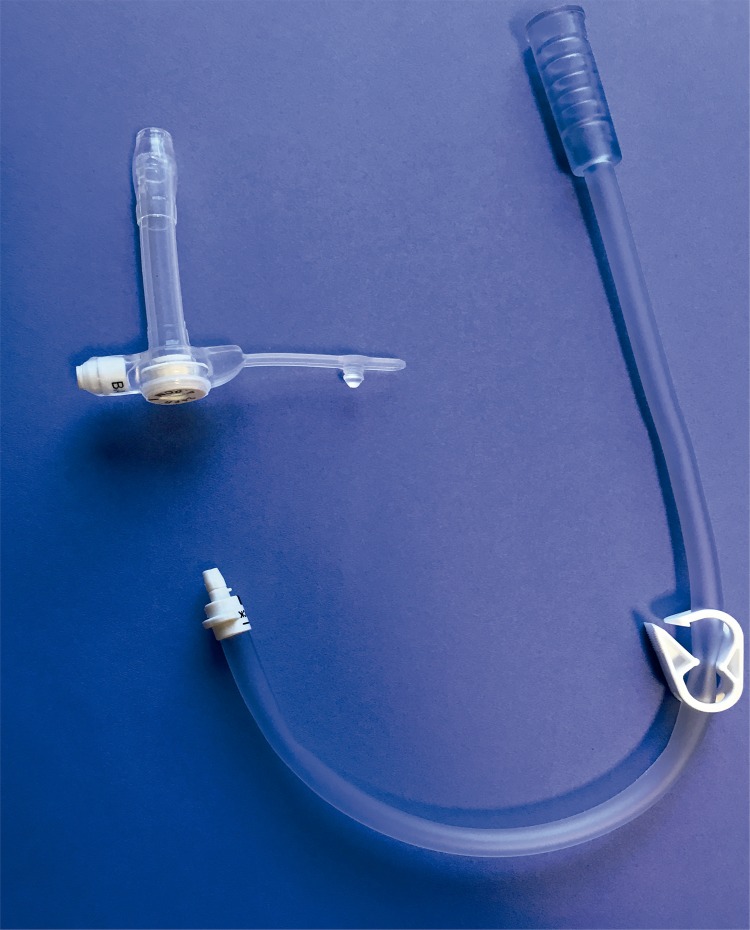
Vesicostomy Button with attachment tubing.

The open placement is performed via 2-3cm incision 2 finger breadths above the pubic symphysis. A cystotomy is created to accommodate an 18-French catheter. The button is then directly inserted into the bladder, the balloon is inflated, and a purse-string absorbable suture is placed to create a snug fit of the detrusor and mucosa around the button. The button is the exact button used for gastrostomy tubes and comes with extension tubing that attaches to the button. To drain the bladder the extension tubing is connected and can drain directly into toilet or urinal (Figure-2).

### Technique for primary placement: Endoscopic/Percutaneous Technique

Patient is positioned into dorsal lithotomy position and the appropriate cystoscope is inserted into the bladder. The dome of the bladder is visualized. A small skin incision is made and a finder needle is inserted followed by a 0.038 wire under direct vision. The finder needle is removed leaving the wire in place; dilators are then passed over the wire to a maximum of 18-French. A vesicostomy button measuring device is used to measure the length of the tract and chose the appropriate button size. The vesicostomy button is placed over the wire under direct vision.

### Placement into an established tract

This technique is used for children with a pre-existing vesicostomy. In the operating room the vesicostomy tract is dilated to 18F and the vesicostomy button measuring device is used to choose the appropriate button size. The button is then placed directly into the vesicostomy tract and the balloon inflated.

After placement it is our practice to continue the button to constant drainage for 24-48 hours then began intermittent bladder drainage via the extension tubing. We change the button every 6-8 weeks in the office with a nurse or by the parent at home.

## RESULTS

Thirteen children have had a vesicostomy button placed at our institution from 2011 to 2015, ages 7 months to 18 years with an average age of 5 years (median 3.8 years). Follow-up has been 24.75 months on average (range 3-54); only 2 patients do not have ongoing urology follow-up as one is followed solely by nephrology and the other has transitioned to adult urology care. Indications for placement included neurogenic bladder (5), non-neurogenic neurogenic bladder disorders (3), and valve bladders (5). Currently 10 children are still using the vesicostomy button while 2 children have had the button removed and are voiding spontaneously and 1 child was converted back to a traditional vesicostomy. All buttons were placed either because patient or family was unable to catheterize secondary to patient anatomy or patient was sensate and did not tolerate CIC. Table-1 shows the patient demographics.

**Table 1:** Patient Demographics.

Patient	Age (years)	Diagnosis	Indications	Placement Technique
1	3	Neurogenic Bladder	Acute Retention, Unable to Tolerate CIC	Existing Vesicostomy
2	3.8	Valve Bladder	Unable to Tolerate CIC, Prior Vesicostomy	Existing Vesicostomy
3	0.6	Neurogenic Bladder	Parents Unwilling to Perform CIC	Endoscopic
4	4.8	Non-neurogenic Neurogenic Bladder	Unable to do CIC due to Anatomy	Endoscopic
5	1.6	Valve Bladder	Placed to Cycle Bladder Pre-transplant	Existing Vesicostomy
6	4.3	Valve Bladder	Parents Unwilling to Perform CIC	Endoscopic
7	13	Neurogenic Bladder	Unable to do CIC due to Anatomy	Existing Vesicostomy
8	5.6	Neurogenic Bladder	Parents Unwilling to Perform CIC	Open
9	5.6	Non-neurogenic Neurogenic Bladder	Patient Unable to Tolerate CIC	Existing Vesicostomy
10	18.3	Non-neurogenic Neurogenic Bladder	Patient Unable to Tolerate CIC, Prior Vesicostomy	Existing Vesicostomy
11	2.8	Valve Bladder	Acute Retention, Unable to Tolerate CIC	Endoscopic
12	11.6	Valve Bladder	Patient Unable to Tolerate CIC, Prior Vesicostomy	Existing Vesicostomy
13	3.1	Neurogenic Bladder	Cloacal Malformation, Patient Unable to tolerate CIC	Endoscopic

Three patients with vesicostomy buttons were transplant recipients. All three children had a diagnosis of a posterior urethral valve. All three patients have normal sensation and had a vesicostomy performed soon after birth for valve and renal failure. Two of the three patients still have buttons in place. The third patient, an 11-years-old, had the button placed for assessment of bladder capacity and possible function as he had had a vesicostomy since birth. However, the patient’s creatinine rose after placement and he was not compliant with drainage schedule, he was converted back to a vesicostomy.

Postoperative complications (within 30 days) were minor including UTI (3), wound infection (1), and button malfunction (3). Button malfunction was considered a complication if the patient was seen in the emergency department or clinic for significant leakage requiring button or tubing change before the planned initial change or for dislodged buttons. Seven patients had the vesicostomy button placed through an existing vesicostomy track at time of vesicostomy revision. Five patients had the button placed via the endoscopic approach. One patient had an open vesicostomy button insertion. Five out of the seven patients (71%) with buttons placed into previous vesicostomy and the one patient (100%) who underwent open placement had leakage around the vesicostomy button. Conversely, none of the patients with the endoscopic approach had leakage.

To assess for upper tract deterioration creatinine and imaging were used if available. Creatinine was measured before and approximately 1 month after button placement in 10 patients. Creatinine remained stable in all 10 patients (maximum change = 0.12 mg/dL). The patient in which the button was removed for non-compliance and creatinine elevation occurred 3 months after placement, this elevation was concomitant with a UTI and resolved with vesicostomy button removal. Upper tract imaging was available in 12 patients. Ten renal ultrasounds confirmed absence of hydronephrosis or stable to improved hydronephrosis post-placement. The other two patents had nuclear medicine scans (MAG3 renal Lasix scan and DMSA) confirming equal and stable function bilaterally.

One patient in our series used the button for bladder cycling prior to successful transplantation.

Four children had recurrent UTI’s documented before insertion and continued to have infections after placement. Three additional children began experiencing UTI’s after placement.

One child presented with erythema at the vesicostomy button site was diagnosed with a wound infection; this was treated with Keflex® for local cellulitis.

Three patients were reported to have button malfunction issues within their first 30 days after placement. These issues included leakage around the button ultimately requiring button revision (1), the button became dislodged requiring nursing replacement (1), and difficulty with the drainage tubing to the button (1) which was resolved with nurse teaching.

Eight vesicostomy patients and 6 CIC patients were compared in regards to QOL using the questionnaire. Mean age of vesicostomy button patient at time of survey was 6.6 compared to CIC group at age 7.3. Table-2 shows patient demographics for both groups.

**Table 2:** Patient Demographics.

CIC Cohort			Vesicostomy Button Cohort		

Age (years)	Sex	Indication for CIC	Age (years)	Sex	Indication for Vesicostomy Button
11	M	Neurogenic bladder	7	M	Valve bladder
7	F	Neurogenic bladder	10	F	Non-neurogenic neurogenic bladder
12	M	Neurogenic bladder	8	M	Neurogenic bladder
7	M	Neurogenic bladder	7	M	Valve bladder
2	M	Neurogenic bladder	4	M	Valve bladder
4	M	Neurogenic bladder	7	M	Neurogenic bladder
			4	M	Valve bladder
			6	M	Non-neurogenic neurogenic bladder

**Figure 3:**
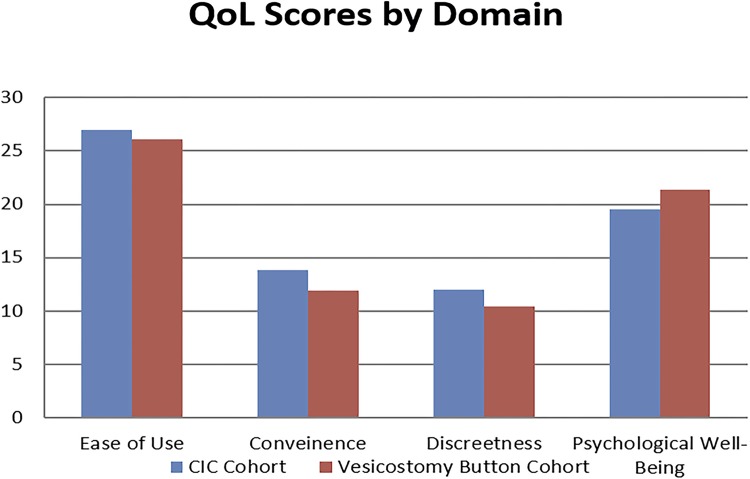
CIC versus vesicostomy QoL scores by domain.

Mean scores were evaluated for each domain and an overall QoL score as shown in Table-3. Figure-3 shows the mean scores for the cohorts in each domain. An overall QoL score included a total of 19 questions with the highest possible score of 95. The vesicostomy button cohort had an average score of 69.8 while the CIC cohort averaged 72.3 (p-value=0.65). Thus, there was no statistical difference in QoL when using the vesicostomy button versus CIC.

**Table 3:** QoL Scores.

	CIC Cohort	Vesicostomy Button Cohort	p-value
Overall QoL Score Max = 95	72.3	69.75	0.65
Mean QoL Score (SD) Max = 5	3.77 (1.47)	3.71 (1.48)	
Overall Ease of Use Score Max = 35	27	26.13	0.88
Mean Ease of Use Score (SD) Max = 5	3.63 (0.51)	3.64 (0.99)	
Overall Convenience Score Max = 15	13.83	11.88	0.19
Mean Convenience Score (SD) Max = 5	4.62 (0.66)	4.19 (0.92)	
Overall Discreetness Score Max = 15	12	10.38	0.20
Mean Discreetness Score (SD) Max = 5	3.99 (0.83)	3.45 (0.69)	
Overall Psychological Well-Being Score Max = 30	19.5	21.38	0.52
Mean Psychological Well-Being Score (SD) Max = 5	3.26 (1.01)	3.6 (0.73)	

## DISCUSSION

The vesicostomy button is an acceptable alternative to traditional vesicostomy or CIC. An important consideration in the use of the vesicostomy button is that is it a temporary measure to allow the child to achieve his/her voiding baseline and develop a definitive plan while keeping his/her upper tracts safe and bladder functioning with social continence. The 30 day morbidity of the button is quite low. Generally, the button is used as a bridge to a more definitive procedure or reconstruction. In our series, the average length of button placement was around 2 years. The plan for the majority of these patients is eventual reconstruction to achieve continence. However, some patients have been very happy with the button and do not desire reconstruction. Because the tube is a vesicostomy button and not a Foley catheter, there is a maximum length of 5 cm and in one larger patient the button required removal because she had grown larger than the maximum offered length.

Urinary tract infections were a prominent finding throughout the study. We found that if the child had recurrent uti’s prior to button placement that this was likely to continue after placement.

Our leakage rate of 71-100% for those patients that had placement into an existing vesicostomy or open button placement was consistent with the findings described by Haider et al. (2). The leakage was significant in 2 patients (40%), these patients eventually required button revision. Endoscopic placement is superior in regards to achieving dryness post-operatively. Existing vesicostomy tract should be avoided if end goal of treatment is total dryness.

We found when reviewing our QoL surveys that the vesicostomy button is comparable to CIC, specifically in regards to ease of use, convenience, discreetness and psychological well-being. Vesicostomy is a good alternative to CIC when it cannot be tolerated due to patient anatomy or intact genital sensation.

To our knowledge, this is the first study to assess pediatric patients’ perceived quality of life while using alternate bladder emptying strategies. Mosiello et al. (7) studied their experience with cystotomy button in the pediatric population and found that it was well accepted by patients and families. While they concluded that it could improve patient satisfaction and quality of life, no objective data was collected. We found both cohorts were satisfied with their quality of life, both had average Qol scores over 3 (3.77 for CIC cohort and 3.71 for vesicostomy button cohort). Satisfactory QoL is a score greater than 2.5 (6).

We look forward to the growing body of literature in regards to vesicostomy button placement and hope to identify those that would benefit the most from its use.

## CONCLUSIONS

We conclude that the button does not decrease UTI’s in the child with a history of UTI’s prior to placement. We also found that endoscopic placement is the best option with the least amount of button leakage. Quality of life is reported as equally good among children doing CIC and using the vesicostomy button for bladder drainage.

## References

[1] 1. Bradshaw CJ, Gray R, Downer A, Hitchcock R. Button vesicostomy: 13 years of experience. J Pediatr Urol. 2014;10:80-7. 10.1016/j.jpurol.2013.06.008 23900025

[2] 2. Haider N, Subramaniam R. Endoscopic insertion of cystostomy button for bladder drainage in children. J Pediatr Urol. 2008;4:457-9. 10.1016/j.jpurol.2008.07.002 18760676

[3] 3. Lacreuse I, Becmeur F, Dheu C, Moog R, Terzic J, Fischbach M. Endoscopic Mic-Key button placement for continent vesicostomy. J Laparoendosc Adv Surg Tech A. 2010;20:297-9. 10.1089/lap.2009.0191 19943778

[4] 4. Bennett S, Bennett S, Bell TE. The gastrostomy button as a catheterizable urinary stoma: a pilot study. J Urol. 2003;170:832-4. 10.1097/01.ju.0000081185.62789.97 12913710

[5] 5. Milliken I, Munro NP, Subramaniam R. Cystostomy button for bladder drainage in children. J Urol. 2007;178:2604-6. 10.1016/j.juro.2007.08.031 17945298

[6] 6. Pinder B, Lloyd AJ, Elwick H, Denys P, Marley J, Bonniaud V. Development and psychometric validation of the intermittent self-catheterization questionnaire. Clin Ther. 2012;34:2302-13. 10.1016/j.clinthera.2012.10.006 23178033

[7] 7. Mosiello G, Lopes Mendes AL, Capitanucci ML, Zaccara AM, De Gennaro M. Button Cystostomy: Is it Really a Safe and Effective Therapeutic Option in Pediatric Patients With Neurogenic Bladder? Urology. 2017;101:73-9. 10.1016/j.urology.2016.09.025 27693876

